# Impact of a randomized possible selves experiment on new retirees’ physical activity and identity

**DOI:** 10.1186/s11556-016-0167-x

**Published:** 2016-08-27

**Authors:** Mélanie G. M. Perras, Shaelyn M. Strachan, Michelle S. Fortier, Brenden Dufault

**Affiliations:** 1 School of Human Kinetics, University of Ottawa, 125 University Private, Ottawa, Ontario K1N 6N5 Canada; 2 Faculty of Kinesiology and Recreation Management, University of Manitoba, Winnipeg, MB R3T 2N2 Canada; 3 George and Fay Yee Centre for Healthcare Innovation, College of Medicine, University of Manitoba, 735 McDermot Ave, Winnipeg, MB R3E 0T6 Canada

**Keywords:** Intervention, Possible selves, Identity, Physical activity, Retirees

## Abstract

**Background:**

Retirement is not always associated with greater engagement in physical activity. Previous interventions informed by possible selves, a type of future-oriented self-representation, proved useful to increase physical activity in young adults. We thus wanted to explore if a similar intervention would yield favorable outcomes in new retirees. We also examined whether possible selves could help increase identity relative to the physical activity context. Identity circumscribes the meanings which help individuals define who they are in a given role (i.e., what it means to be a physically active person). The strength of identification as a physically active person increases when individuals endorse these meanings more strongly. Possible selves may be tied to identity as they allow individuals to imagine themselves as physically active, which has been argued to incite changes to one’s sense of self. Hence, the overall aim of this study was to determine whether a possible selves intervention would increase physical activity behaviour and physical activity identity in a group of newly-retired individuals.

**Methods:**

A total of 294 participants were randomized into one of three groups: (a) a repeated group with three possible selves image generation exposures, (b) a one-time group with one possible selves image generation exposure, or (c) a control group. Participants completed self-report measures at baseline and follow-up assessments were taken at weeks 4, 8, and 12 of the study. The measures for the outcomes of interest were the Godin Leisure Time Exercise Questionnaire and the modified Exercise Identity Scale.

**Results:**

Repeated measures mixed-effects models analyses with maximum likelihood estimation revealed no significant differences between groups on physical activity behaviour (*p* = 0.34) or physical activity identity (*p* = 0.97) at follow-up time points. However, a time effect was found for physical activity (*p* <.01) and physical activity identity (*p* <.01), which increased across time (baseline-to-12-week follow-up) in all three groups. Such a time effect (inconsequential to group assignment) suggests that the observed increases in physical activity and identity cannot be attributed to an exposure to a possible selves intervention.

**Conclusions:**

While the intervention failed to significantly increase physical activity identity and physical activity in newly retired individuals, we suggest future research directions for interventions targeting new retired individuals.

## Background

### Retirement and self

From mid-to-late life, people’s physical activity levels decline [1]. This decrease may result in older adults missing out on the benefits associated with regular physical activity [2]. Retirement may present older adults with an opportunity to thwart these age-related declines by affording them increased time to be physically active [3, 4]. However, research examining the association between retirement and self-reported physical activity reveals that for many retirees, this life transition does not lead to increased physical activity [5–8].

Researchers highlight the need to understand the “personal factors” [8] related to physical activity among retirees as they could be employed in interventions aimed at helping retirees transition into a physically active retirement [8, 9]. *Self-perceptions* are a category of personal factors that are central in understanding physical activity behaviour and behaviour change [10, 11] and may be especially relevant to new retirees. Indeed, the transition from work to retirement is viewed as a period for self-exploration and enhancement during which retirees may self-reflect on their health and who they are (e.g., [12–14]). As such, we turn to one type of self-perceptions: possible selves.

### Possible selves

Possible selves are future-oriented self-representations which circumscribe the thoughts people have about their future and their potential related to specific roles [15]. Since possible selves are anchored in the future - and thus are not confined by what is factual in the present – they represent malleable elements of the self [16]. Possible selves are not just *any* aspect of one’s imagination, rather they usually hone in on individuals’ enduring hopes and fears for the future [15]. As per these authors, possible selves provide motivation and guidance for change. First, they provide a vivid snapshot of what is possible for someone (good or bad) which serves as a goal to be pursued or avoided. Secondly, possible selves provide a context to evaluate or interpret current behaviour.

### Possible selves and physical activity

The possible selves construct has been employed in physical activity/exercise research. A cross-sectional study by Harju and Reed [17] found, in a sample of undergraduate students, that the supposed attainment of a possible self in the exercise purview positively coalesced with exercise behaviour, fitness, and self-efficacy for exercise. Additional cross-sectional findings from Whaley [18] demonstrate how the reporting of possible selves pertaining to body image can distinguish inactive and active, middle-age women. Furthermore, Whaley and Schrider [19] showed that older adults participating in a 10-week exercise program had well-articulated possible selves related to physical activity. Recently, possible selves have been assessed relative to the physical activity levels of new retirees [20]; these authors confirmed that newly retired individuals’ possible selves pertaining to physical activity were positively associated with concurrent physical activity. And this has been recently confirmed in a prospective study [21]. As such, physical activity possible selves appear to be positively related to aspects of physical activity.

Possible selves have also been leveraged in *interventions* designed to increase physical activity. Ouellette, Hessling, Gibbons, Reis-Bergan, and Gerrard [22] found that university students who thought and wrote about their exercise participation from a future perspective (i.e., possible self) reported increased exercise behaviour four weeks later. Subsequently, Murru and Martin Ginis [23] found that inactive university students who read a script to help generate images of themselves in the future as either an exerciser or an inactive person reported greater physical activity four and six weeks post manipulation than participants in the control group. Similarly, Strachan, Marcotte, Giller, Brunet and Schellenberg [24] randomized insufficiently-active adults (aged 18–64) to either a self-enhancing possible selves intervention (consistent with [23]) or an enhanced “self-regulatory” possible selves intervention (participants’ possible self image included the self-regulatory steps they would take to achieve their possible self). Participants in both conditions reported significantly higher physical activity than their control counterparts (at 4- and 8-week follow-up for the self-regulatory condition and at 8 weeks for the self-enhancing condition). These studies suggest that a *one-time* focus on images, including possible selves, can impact physical activity behaviour. Given the promising results of these studies, a possible selves intervention may represent a reasonable tool through which we can attempt to increase physical activity among retirees and by the same token, heed recommendations by Ouellette et al. [22] to examine the impact of such interventions on older adults.

### Possible selves and identity

While possible selves interventions can lead to increases in physical activity, they also hold the potential to have another important impact, that of increasing the extent to which individuals identify with physical activity *in the present* (physical activity identity). Indeed, researchers argue that the consideration of possible selves can affect and lead to enduring changes in current self-views (i.e., identity; [15, 25, 26]). Given that Markus [27] and Burke [28] have both commented that identities are resistant to change, individuals may require *repeated* occasions to reflect on a physically-active possible self for identity to be impacted. This assertion is consistent with that of Hoyle and Sherrill [25] who argue that the *chronic* activation of a possible self can impact current self-representations (i.e., identity). No research to date has examined the effects of a physical activity possible selves intervention on physical activity identity or considered whether repeated exposure to the possible self image is necessary for a change in identity to occur.

If imagining oneself as a physically active retiree could encourage people to identify with physical activity, this consequence may represent an important way in which sustained physical activity behaviour change could be fostered [29]. Indeed, the ameliorative influence of identities on physical activity/exercise is well documented (for a review, see [30]). Strength of exercise identity also positively relates to self-efficacy for exercise, exercise frequency, and the articulation and carrying out of exercise intentions [31–33]. Furthermore, the positive relationship between physical activity identity and related behaviour is demonstrated in middle-aged and older adults [34–36] as well as in recent retirees [20, 21]. Therefore, possible selves interventions may lead to changes in physical activity identity, a known correlate of physical activity behaviour. Further, testing the utility of possible selves interventions at increasing identity should also be of interest to scholars interested in identity change.

### Experimental purposes and hypotheses

The overall aim of this experiment was to determine if a possible selves intervention would increase physical activity identity and physical activity behaviour in a group of newly-retired individuals. One specific aim was to compare physical activity levels and identity across three conditions of an experimental design: a *repeated* possible selves intervention, a *one-time* possible selves intervention, and a control group. We hypothesized that participants in the *repeated* possible selves group would report higher levels of physical activity and identity over the course of the intervention than the *control* group (hypothesis 1) and the *one-time* possible selves group (hypothesis 2). The *one-time* condition was expected to report higher levels in physical activity identity and physical activity over the course of the intervention than the control group (hypothesis 3).

## Methods

### Participants

A total of 294 newly retired men and women were randomized in the present study. Specific inclusion criteria included confirmation of retirement status (i.e., collecting pension/retirement-like income, and considering oneself “retired”) and confirmation of current health status (allowing for increased physical activity). To allow for a focus on *newly-retired* individuals, we restricted eligibility to retirees within three years of retirement. This cut-off is consistent with past research on new retirees’ leisure experiences (e.g., [37]). Further, we focused on recently-retired, as opposed to all retired individuals, based on the assumption that the physical activity patterns and self-perceptions related to this role of newly-retired individuals should be less entrenched than those of more “seasoned” retirees. Moreover, individuals exercising four times per week or more or reporting a strong physical activity identity were excluded as they were deemed not likely to benefit from the intervention.

The average age of the participants in the sample (i.e., across conditions) was 63.4 years old. The majority of the sample was Caucasian (94 %), female (68.5 %), married (60 %), and highly educated (with almost 30 % of participants reporting having a degree higher than a bachelor’s degree). The body mass index (BMI) was calculated with self-reported weight and height data; the BMI for the whole sample was 29.74, corresponding to the overweight classification [38]. We present sociodemographic information per each condition in Table 1.

**Table 1:** Participant demographics at randomization (*N* = 294)

	Group		
Characteristic	Control (*n* = 88)	One-time (*n* = 107)	Repeated (*n* = 99)
Age, mean (*SD*)	63.02 (4.84)	63.84 (4.59)	63.23 (4.71)
Sex^a^			
Women	65 (74.7)	70 (66.0)	63 (65.6)
Men	22 (25.3)	36 (34.0)	33 (34.4)
Ethnicity^b^			
Caucasian	86 (97.7)	94 (87.9)	95 (97.9)
Other	1 (1.10)	6 (5.5)	1 (1.1)
Aboriginal/Native	0 (0.00)	4 (3.7)	1 (1.00)
South Asian	1 (1.1)	3 (2.8)	0 (0.00)
Marital status^c^			
Married	45 (52.30)	67 (63.2)	63 (64.9)
Divorced/Separated	16 (18.6)	15 (14.1)	15 (15.4)
Living w/ partner	9 (10.5)	11 (10.4)	7 (7.2)
Single/Never married	10 (11.6)	7 (6.6)	6 (6.2)
Widowed	6 (7.0)	6 (5.7)	6 (6.2)
Education^d^			
University: above B.A.	28 (32.2)	30 (28.0)	26 (26.5)
University: B.A.	18 (20.7)	26 (24.3)	23 (23.5)
College/CEGEP	22 (25.3)	17 (15.9)	14 (14.3)
No post-secondary	8 (9.2)	19 (17.8)	17 (17.3)
Other	11 (12.6)	15 (14.0)	18 (18.3)
Body mass index^e^, mean (*SD*)	29.98 (6.37)	28.73 (5.98)	29.97 (5.63)
Retirement^f^, y, mean (*SD*)	1.86 (1.15)	1.77 (1.45)	1.63 (1.38)
Perceived health^g^, mean (*SD*)	6.87 (.99)	7.09 (.88)	6.77 (.98)
CFC^h^, mean (*SD*)	3.37 (.56)	3.61 (.62)	3.52 (.56)
Imaging ability^i^, mean (*SD*)	34.64 (7.06)	35.70 (6.87)	33.42 (8.87)

Data are presented *Count (%)* unless indicated otherwise. ^a^ Five participants did not report their gender; 68.5 % of the sample was female overall. ^b^ Two participants did not report their ethnicity; 94.2 % of the sample was Caucasian overall. ^c^ Five participants did not report their marital status; 60.6 % of the sample was married overall. ^d^ Two participants did not report their education; 28.8 % of the sample had a degree higher than a BA. ^e^ Two participants did not report their height. The BMI of the sample overall was 29.74 (*SD* = 6.43). ^f^ The participants in the sample were retired for 1.75 years on average. ^g^ Eight participants missed one of three items which comprises the scale. Scores could range between 3 and 10. The average for the sample was 6.92 (*SD* = .96) overall.^h^ Consideration of future consequences (CFC). Scores could range between 1 and 5. The average for the sample was 3.51 (*SD* = .59) overall. ^i^ Scores could range between 0 and 44. The average for the sample was 34.62 (*SD* = 7.69) overall

### Procedures

All procedures were approved by the appropriate Research Ethics Board. In an effort to reach as many retirees as possible, we decided to deliver the intervention online through a web survey platform. In the health purview, internet-delivered interventions carry some notable advantages over their lab-based counterparts (for a discussion of these advantages, please see [39, 40]). Furthermore, a growing body of research is showing that internet-delivered interventions are acceptable and/or effective in increasing physical activity in older adults [41–46].

Participants were recruited via word-of-mouth, social media and online postings, local advertisements (i.e., public libraries and notice boards, newspaper), newsletters from organizations catering to older adults, and e-mail lists. All advertisements featured the secure URL link for the online survey platform (powered by Fluid Surveys). Interested individuals could visit the platform to determine eligibility, and if eligible, read and provide informed consent to participate in the study, and complete baseline measures. Following completion of baseline measures, participants were randomly assigned to one of three conditions: control (*n* = 88), one-time intervention (*n* = 107), and repeated intervention (*n* = 99). Randomization took place online via a single multiple choice question with nonsensical answer choices; each choice was tied to a different group assignment. To minimize order bias, the answer choices were shown in a random order every time the page was viewed. Follow-up data were collected at four, eight, and 12 weeks after baseline. At all times and for each follow-up time point, participants had the option of saving their unfinished questionnaire and completing it later (by e-mailing themselves a link to retrieve the questionnaire bookmarked at the last completed page).

### Intervention

#### One-time possible selves intervention condition

Immediately after completing baseline measures, participants randomized to the *one-time* possible selves intervention group completed a standard image generation task adapted from Murru and Martin Ginis [23]. Participants were invited to watch a short video embedded within the web survey platform. Before clicking on the video to start, participants were reminded to turn up the volume of their device since the video (through JavaScript) could not be stopped, paused, fast-forwarded, or played anew. Participants were also reminded of the definition of moderate-to-vigorous physical activity given its reference in the video.

The video was comprised of a series of slides (i.e., Powerpoint slides saved as a Windows Media Video file) with text presented against a solid background. A narrator also read the text as follows:

The following video is a very important component of the present study. We kindly ask you to watch and listen carefully. It is only a few minutes long. This video addresses how you see yourself in the future. We all think about the future to some extent. When doing so, we usually think about the kinds of experiences that are in store for us and the kinds of people we might possibly become. We are interested in your impression of yourself five (5) to ten (10) years from now. More specifically, we would like you to think about yourself in the future as a physically active retiree. You incorporate physical activity to your lifestyle on most days of the week at a moderate to vigorous intensity. Five (5) to ten (10) years from now you have the energy to carry out your daily tasks and all your personal goals for retirement. When you think about yourself five to ten years from now as a retiree who is physically active on a regular basis, what images come to mind? Please take a few minutes to imagine and think about this image before moving on to the next page. On the following pages, you will be asked to answer some questions about this image.

This part of the intervention (text/narration) was 2.5 min long. Through presenting the text via video/narration we sought to solicit the participants’ attention and immersion in the image generation task (and not just a quick skimming of the text). Once the sentence *Please take a few minutes to imagine and think about this image* appeared on screen, participants were given two minutes to carry out this imagining/thinking task after which the screen turned black, prompting participants to move ahead to the next page. In all, the image generation task (intervention) took about 4.5 min. The task heeds recommendations advocated in other literature as per the ideal run time (i.e., five minutes) for a video component in an Internet-delivered intervention [47].

Following this task, and in line with Murru and Martin Ginis [23], participants answered seven open-ended questions to ensure elaboration upon the image (i.e., image’s appearance, energy level, attitude toward life, general health, relationships, achievements and any other comment/thought that came to mind), which also served as a manipulation check for the image generation task. Further, “compliance” checks were conducted by asking two questions about the content of the video (to confirm viewing) and by asking if (and to what extent) participants used the time provided to think about the image of themselves as a physically active retiree.

#### Repeated possible selves intervention condition

Participants randomized to the *repeated* intervention group completed the standard possible selves image generation task (as described above) on three separate occasions, each one week apart. The three intervention exposures were practically identical with the exception of a few minor changes made to the video (i.e., change in background colour, narrator) and to the compliance checks (different “content” questions were posed). All told, participants in the repeated condition completed the subsequent intervention exposures before the week 4 (time 2) follow-up data collection point.

#### Control condition

During the 12-week study duration, participants randomized to the *control condition* only completed the follow-up measures at four, eight, and 12 weeks. The participants did not receive any intervention-like materials. The “no treatment” option was predicated on arguments that finding intervention effects can be more difficult when an active comparator (i.e., some form of intervention) is given to control participants [48].

### Measures

#### Eligibility measures

Eligibility was assessed and instantaneously determined online via the Fluid Surveys platform; we opted for one-item eligibility measures of physical activity and physical activity identity. The multi-item measures used during the actual intervention (described below) were not helpful for eligibility purposes because of the platform’s inability to calculate means or scores.

##### Physical activity

Prospective participants’ physical activity level was assessed via a single item consistent with Godin and colleagues’ work [49–51]. The item read as follows: *How often have you participated in one or more moderate-to-vigorous physical activities for at least 30 min in one day during your free time in the last three months?* Definitions of leisure time, moderate and vigorous physical activity were provided. Seven answer choices were displayed and ranged from: *Never* to *four times or more per week*. Potential participants selected one answer choice; those reporting exercising four times per week or more were excluded.

##### Physical activity identity

Prospective participants’ physical activity identity was assessed via a single item recently adapted for physical activity research by Carraro and Gaudreau [52] but originally developed by Aron, Aron, and Smollan [53]. Potential participants were shown a figure with seven pairs of increasingly intertwined circles numbered from 1 (low identification) to 7 (high identification) and were asked to select the number that corresponded to their relationship with physical activity (i.e., the extent to which they think physical activity is a part of who they are). Potential participants who selected 6 or 7 were excluded.

#### Main measures

The main measures of the study are described below.

##### Sociodemographic information

Information on age, sex, ethnicity, marital status, education level, body mass index, years in retirement, and perceived health [54] was collected and is presented in Table 1.

##### Godin Leisure Time Exercise Questionnaire (GLTEQ)

Using the GLTEQ [55] participants indicated the number of bouts of *15 min* or more of physical activity performed at strenuous and moderate activity performed over a 7-day period. The GLTEQ is valid [56] and has been used in older adults [36, 57]. To encourage more accurate reporting, we included additional instructions that normalized the difficulties of being physically active and encouraged honest reporting as recommended and proven effective by Gagné and Godin [58]. For the present study, bouts of moderate and vigorous activity were summed to obtain moderate-to-vigorous physical activity bouts. Physical activity was assessed at baseline, 4, 8, and 12 weeks.

##### Physical activity identity scale

A slightly modified version of the Exercise Identity Scale (EIS; [59]) was used to assess physical activity identity. The original scale comprises nine items and uses a 7-point Likert-type scale ranging from 1 (*strongly disagree*) to 7 (*strongly agree*). Sample items include: *I consider myself an exerciser* and *When I describe myself to others, I usually include my involvement in exercise*. The modifications made to the scale were consistent with Strachan et al. [36] in that the word “exercise” was exchanged for “physical activity” (e.g., *I consider myself a physically active person)*. This change is based on research suggesting that older adults find the term “physically active person” more self-descriptive than “exerciser” [36, 60]. All other aspects of the original scale were retained. Anderson and Cychosz [59] reported a strong Cronbach’s alpha (α = .94) and test-retest reliability for the original version of this scale (r = .93). The modified scale used by Strachan et al. [36] also proved reliable (Cronbach’s α of .90). In this study, items of the scale proved reliable at all four time points – baseline, 4, 8, and 12 weeks. (α’s: .87; .88; .91; .91, respectively).

#### Covariates

Given the temporal (i.e., oriented toward the future) and imaginal (i.e., image generation task) aspects of the intervention, covariates pertaining to consideration for future consequences and imaging ability were entered in the analyses.

##### Imaging Ability Questionnaire (IAQ)

The *image generation* subscale of the IAQ [61] was used to assess how vividly people generate images. The full IAQ (with *absorption* subscale; 21 items) boasts good internal consistency (α = .93) and test-retest reliability (r = .92). The IAQ has been used in exercise imagery interventions as a control variable (e.g., [62]) and was used in this capacity in the present study. Participants indicated, on 11 items, the extent to which they can generate certain images (e.g., *imagine clouds with a storm blowing up and flash of lightning*) using a 5-point scale ranging from 0 (*no image at all*) and 4 (p*erfectly clear and vivid*). The image generation subscale was reliable in the present sample (α = .91). Imaging ability was entered as a covariate in the analyses.

##### Consideration of Future Consequences (CFC)

The 12-item CFC scale [63] was used to measure the extent to which individuals consider the immediate and distal consequences of their actions and was included as a covariate in the analyses. The scale has been validated previously in a number of samples (α’s ranging between .80 and .86; test-retest reliability between .72 and .76). CFC has been previously shown to influence a possible selves intervention targeting physical activity [22]. This scale features a Likert-type scale which ranges from 1 (*extremely uncharacteristic*) to 5 (*extremely characteristic*). A sample item reads: *I think it is important to take warnings about negative outcomes seriously even if the negative outcome will not occur for many years.* The scale was reliable in the present sample (α = .78).

### Statistical analysis

#### Repeated measures

In order to test for significant changes in physical activity identity and physical activity between conditions and over time (baseline, 4, 8, and 12 weeks), we conducted repeated measures mixed-effects models analyses with maximum likelihood estimation using PROC MIXED of SAS for Windows version 9.3 (SAS Institute Inc., Cary NC). The decision to run these models was predicated on the nested nature of the data (i.e., time points nested within participants), which introduces within-subject correlation between repeated measures of the outcome. Mixed-effects models allow for statistical dependence between observations within subjects and incorporates it into the model estimation algorithm, thereby providing unbiased effect estimates and appropriate standard errors. Furthermore, mixed models are increasingly used in longitudinal studies as they allow the inclusion of participants with missing data [64]. As a result, all participants who, at minimum, completed the baseline measurement and the first follow-up measurement (i.e., time 2) were included in the analyses (*n* = 221). The three conditions (groups), time, and condition by time interaction were modeled as fixed effects. The two covariates – consideration for future consequences and imaging ability – were also entered as fixed effects. Physical activity identity was modeled as continuous using linear mixed-effects with an unstructured covariance type. Physical activity (bouts) is a count variable however, and was modeled via negative-binomial generalized linear mixed-effects with an autoregressive covariance structure. Time was modeled as a categorical effect. Various within-subjects correlation structures were explored for each model, beginning with an unstructured approach, which allows all correlations to be estimated freely. More parsimonious correlation structures were then compared via likelihood ratio tests, and on grounds of parsimony were chosen if they did not perform significantly worse than the more intensive unstructured approach. The significance level for tests was set at *p* < 0.05.

#### Sample size calculations

Sample size calculations were performed in G*Power [65]. A minimum sample size of 66 per group is needed to detect a small effect size (d = .20) at .80 power and at an alpha level of .05. This sample size is predicated on a repeated measured study design with three groups (i.e., control, one-time, and repeated) which are assessed at four time points (i.e., baseline, 4, 8, and 12 weeks). Given the inclusion of two covariates (i.e., CFC and imaging ability), a sample size of 80 is preferred. The present study was sufficiently powered to detect differences between groups across time.

## Results

### Retention and attrition analysis

**Fig. 1:**
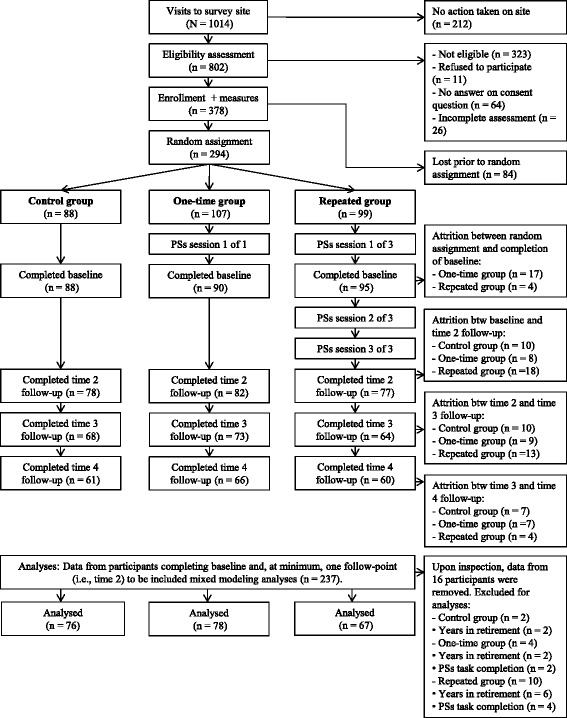
Flow Chart of the Study. The exclusion of data for analytic purposes was predicated on participants’ reported date of retirement (i.e., exceeding three years; ten participants) and unsatisfactory completion of the possible self task/s (six participants)

After completing eligibility, consent and baseline measures, a total of 294 participants were randomized into one of the three groups. The detailed flow chart of the study is featured in Fig. 1. We note that 21 participants chose to leave the study before completing post randomization activities related to the baseline time point. As such, 273 participants fully completed the baseline time point. At time 2 follow-up (four weeks post baseline), 237 participants continued participation and had filled out time 2 measures. At time 3 (eight weeks post baseline), 205 participants completed measures. Finally, 187 participants completed the final follow-up measures at time 4 (12 weeks post baseline). From randomization through to time 4 follow-up, we retained 63.61 % of the sample. There were no significant differences in socio-demographic variables (as itemized in Table 1) between participants who completed the entire study (*n* = 187) and participants who dropped out (anytime after randomization; *n* = 107). Attrition was not influenced by group assignment. Lastly, the completion of follow-up possible self image generation tasks for the “repeated” group was satisfactory. The second task was completed by 88.57 % of participants. The third and final task was completed by 72.86 % of participants.

### Manipulation checks

A manipulation check was conducted on the data from participants in the two intervention conditions. The first and second authors conducted a review of participants’ answers to manipulation check questions described previously. They checked for non-responses and appropriateness of answers (i.e., comments on point). Further, the same authors looked at the “compliance” questions to determine if participants watched the video(s) and used the time provided to think about the image of themselves as a physically active retiree. These steps prompted the removal of all data for six participants due to lack of compliance with the experimental protocol. Further data inspection prompted the removal of all data for 10 participants by reason of ineligibility (i.e., years in retirement). As a result, the removal of 16 participants led to a final analytic sample of 221 participants, split across groups as follows: 76, control; 78, one-time, and, 67, repeated.

### Data management

Prior to running the repeated measures analyses, patterns of missing data and data distribution were examined. We examined missing data against group assignment itself and all sociodemographic variables presented in Table 1. Through the expectation maximization algorithm of the missing data package in SPSS version 22, it was determined that missing data were missing completely at random (MCAR). No data distribution issues were detected.

### Intervention outcomes

To test the hypotheses that the *repeated* possible selves group would report higher levels of physical activity identity and physical activity over the course of the intervention than the *control* group (hypothesis 1) and the *one-time* possible selves group (hypothesis 2), and that the *one-time* group would too report higher levels and greater changes in physical activity and physical activity identity over the course of the intervention than the control group (hypothesis 3), we ran mixed models analyses with restricted maximum likelihood estimates for the effects of time, group assignment, and the group*time interaction set as fixed factors. A statistically significant group*time interaction would indicate changes in outcomes over time are different in the three groups. Consideration for future consequence and imaging ability were entered as covariates.

#### Physical activity identity

For the physical activity identity outcome, an unstructured covariance matrix proved to be the best fit. No group*time interaction was found; only a time effect was detected (see Table 2). Simply put, the three groups displayed similar increases in their physical activity identity over time; the increase, however, is not attributable to group assignment (given the lack of interaction). The means along with confidence intervals for each group at every time point are also presented in Table 2. Analyses showed that CFC had a significant positive influence on the prediction of physical activity identity (*p* < .01; estimate = .62 [95 % CI 0.34, 0.89]). Imaging ability was not significantly related to the outcome (*p* = 0.57; estimate =-.07 [−0.30, 0.17]).

**Table 2:** Means and 95 % CI for three intervention groups (analytic sample *N* = 221)

Outcome measure	Control *n* = 76 (mean and 95 % CI)	One-time *n* = 78 (mean and 95 % CI)	Repeated *n* = 67 (mean and 95 % CI)	Repeated measures model
PA Identity				
Baseline	3.82 (3.51–4.13)	3.68 (3.40–3.97)	3.69 (3.37–4.00)	↑ over time in all groups
4 weeks	3.95 (3.66–4.25)	4.01 (3.74–4.29)	4.13 (3.83–4.43)	Time (T): *p* <.01
8 weeks	3.96 (3.63–4.28)	4.01 (3.70–4.32)	3.89 (3.55–4.23)	Group (G): *p* = 0.97
12 weeks	4.32 (3.99–4.64)	4.16 (3.85–4.47)	4.20 (3.86–4.53)	T x G: *p* = 0.39
PA (bouts)				
Baseline	2.41 (1.73–3.37)	3.31 (2.46–4.44)	2.87 (2.07–3.99)	↑ over time in all groups
4 weeks	3.48 (2.56–4.72)	4.87 (3.68–6.45)	4.23 (3.12–5.74)	Time (T): *p* <.01
8 weeks	3.61 (2.64–4.94)	4.53 (3.36–6.12)	3.67 (2.62–5.13)	Group (G): *p* = 0.34
12 weeks	3.86 (2.79–5.34)	4.29 (3.15–5.83)	3.87 (2.77–5.41)	T x G: *p* = 0.97

*PA* physical activity; PA identity scores vary between 1 and 7. Covariates included: Consideration for future consequences (CFC) and imaging ability. For both PA identity and PA, CFC was significant (*p* <.01, *p* = 0.04, respectively)

#### Physical activity

For this outcome, negative binomial distribution proved the best fit for the count data (i.e., bouts of physical activity). Again, no group*time interaction was found; only a time effect was detected (see Table 2). The three groups displayed similar increases in their physical activity over time; the changes, however, are not attributable to group assignment (given the lack of interaction). The means along with confidence intervals for each group at every time point are presented in Table 2. The prediction of physical activity was also significantly influenced by CFC (*p* = .04; estimate = .25 [95 % CI .01, .50]). Imaging ability was not significantly related to the outcome (*p* = 0.07; estimate = −.19 [95 % CI -.39, .02]).

## Discussion

The aim of this study was to determine whether a possible selves intervention would increase physical activity behaviour and identity in a group of newly-retired individuals. Using an experimental design, we compared changes in identity and physical activity across time and between three conditions: a *one-time* possible selves intervention, a *repeated* possible selves intervention, and a control group. We hypothesized that differences over time in identity and physical activity would be found between participants in the *repeated* possible selves group as compared to the *control* group (hypothesis 1) and the *one-time* possible selves group (hypothesis 2). Participants in the *one-time* group were expected to report greater differences over time in both outcomes when compared to the control group (hypothesis 3). All hypothesized group differences did not materialize. However, over time, all three groups reported marginally higher levels of identity and physical activity irrespective of group assignment.

### Effects of the possible selves intervention on physical activity identity

While identities are known to be rather stable (e.g., [28]), other authors [11] have argued that individuals can experience shifts and changes in self-views, sometimes in a matter of days. Further, Hoyle and Sherrill [25] argue that recurrently thinking about a possible self and its consequences can bring about changes in self-representations. From this theoretical perspective, we speculated that possible selves might bring about changes in identity and that repeated possible selves activation should have the greatest impact on identity. One possible interpretation of our findings of null effects of the intervention on identity is that a physical activity possible selves intervention is not an effective way to increase identity. Considering that no other possible selves intervention, to our knowledge, has attempted to increase identity in the physical activity purview, dismissing the possibility seems premature. Perhaps a hybrid and multifaceted approach is needed to truly impact identity. We offer a few possibilities of such approaches.

An imagery intervention conducted by Cooke, Duncan, Hall, and Rodgers [unpublished observations] shares with our study, the idea that the act of imagining oneself as an exerciser can be a fruitful way to increase identity. In their 36-week exercise program for female exercise initiates, individuals who underwent an exercise imagery intervention in addition to exercise reported significantly stronger exercise role identity at 9 weeks than those within an exercise plus attention control condition. The findings from this study lead to two pertinent observations. First, the guided imagery intervention used in the Cooke et al. study and our possible selves image generation task shared similarities in that both involved evoking images of the self as an exerciser. Giacobbi et al. [66] have even commented on the close ties between mental imagery and possible selves. However, the imagery intervention used by Cooke et al. (i.e., script and lab procedures) provided more elaborate and multifaceted imagery sessions (e.g., more frequent exposure; conducted within the laboratory) than the present possible selves protocol; a more elaborate possible selves image generation task may have led to increases in identity in our study. Second, the study by Cooke et al. included, in addition to the imagery intervention, a physical activity component. As noted by Cooke et al., and others (e.g., [27, 67]), physical activity participation alone may not coalesce with increased identity and the addition of the imagery component in the study by Cooke et al. appeared to help increase identity. Indeed, previous research has pointed to the likely reciprocal relationship between physical activity and identity [19, 52, 60, 68]. Increases in identity are often tied to greater behavioural output - wherein physical activity levels increased significantly. Herein, the image generation tasks were not part of an exercise intervention or program. Future research should consider whether a possible selves intervention combined with a physical activity intervention/program will yield gains in identity and which one has the greatest effect.

### Effects of the possible selves intervention on physical activity

Markus and Nurius [15] argue that possible selves encourage behaviour change by providing motivation and guidance in the form of standards for comparison. Indeed, possible selves have been used with success in a few physical activity/exercise interventions [22–24]. We are therefore surprised that, in the present study, participants exposed to a physical activity possible selves intervention did not report more physical activity than controls.

Methodological considerations offer potential explanations for why we failed to find group differences in physical activity patterns, where others did. Ouellette et al. [22] found that university students who participated in a exerciser possible selves intervention, and scored high on consideration of future consequences, increased exercise behaviour at four weeks post intervention. However, their study did not include a control group, so it is unclear if the effect is attributable to the intervention. Interestingly, without a control group, our findings would also suggest an effect of the possible selves interventions, as we too found changes in physical activity amongst all participants. Murru and Martin-Ginis [23] found that university students exposed to a possible selves image generation task exercised more at 4 and 8 weeks follow-up than those exposed to a control activity. These researchers employed a more stringent eligibility criterion than we did (i.e., less than three versus less than four 30 min bouts of physical activity per week). Our slightly more active participants had less room to increase their physical activity making it more difficult for us to detect a change due to the intervention. Recently, Gunnell, Crocker, Mack, and Zumbo [69] also failed to find a possible selves intervention effect on physical activity in a community sample in which no limits were imposed on baseline physical activity levels. These findings suggest that physical activity possible selves interventions may work best for those who are inactive. Finally, in a study by Strachan, Marcotte, Giller, Brunet and Schellenberg [24] we note that participants were instructed to read the Canadian Physical Activity Guidelines [70] prior to partaking in one of two possible selves image generation tasks. Perhaps the exposure to the physical activity guidelines (i.e., messaging), coupled with the future perspective of the PSs task (i.e., seeing oneself engaging in regular physical, five to ten years from now) highlighted participants’ discrepant physical activity behaviour (or lack thereof), which in turn galvanized their behaviour. The purported impact of being presented with physical activity guidelines is, of course, speculative.

The finding that *all* participants reported increased physical activity suggests that we cannot attribute physical activity increases to exposure to a possible selves intervention. All participants may have been disposed to a form of measurement reactivity [71]. For example, participants likely engaged in self-monitoring through self-reporting their physical activity at several time points via a questionnaire. If participants noticed low or reduced physical activity levels, this awareness may have energized them to increase their physical activity. In a similar vein, having participants complete questionnaires about physical activity (e.g., their intentions, thoughts, etc.) may have increased their chance of performing that behaviour – something known as question-behaviour effect [72]. This effect, documented in the physical activity/exercise purview, operates by making individuals’ underlying attitudes about a particular behaviour more accessible which, in turn, can lead to the behaviour being enacted [73, 74]. Our questionnaires, asked of all participants, included such measures. Considering that we cannot attribute the sample-wide increase in physical activity to our intervention activities, we speculate that some of these measurement aspects may be responsible for the small increase in physical activity reported across our sample.

When considering the null results for *both* identity and physical activity, we ponder whether our approach, while inspired by previous studies, was appropriate for newly retired individuals. Hooker [75] opined: “Goals in later life may be less normatively structured than early and midlife [… and as such] their (older adults’) activities are the most likely to be motivated by their own personal agendas” (p. 111). Herein, and in line with past studies targeting younger adults (e.g., [22–24]), we utilized the same, broadly-defined possible self (i.e., physically active retiree) across groups partaking in the image generation tasks. Perhaps the one-size-fits-all nature of our intervention materials lacked personal meaning, thus dissuading participants from engaging fully in the tasks. Two recent reviews on the features of effective physical activity interventions in older adults [76, 77] point to the importance of personalizing of interventions. In the possible selves purview, this suggestion could take the form of allowing participants to articulate and or revise [16] their own physically active possible self for retirement. Such an individualized approach may be appropriate for retirees for whom generalized possible selves may lack relevance. In the same vein, McDonald, O’Brien, White, and Sniehotta [78] further confirm that “routes to retirement are highly individualistic”. Engagement in physical activity may thus be influenced by personal preferences, obligations, and other competing goals. Therefore, personalized interventions may prove more helpful in getting retirees physically active. Finally, we cannot dismiss the possibility that image generation may become more difficult for older adults. In their article on motor and exercise imagery, Kalicinski and Lobinger [79] review mixed evidence linking motor imagery ability deterioration due to age. However, it is “unclear whether this deterioration is selectively influenced by age” (p. 66). For now, though, linking age and difficulty in imagery tasks in the exercise purview is quite speculative.

### Strengths and limitations

The present study draws strength from its innovation. The study was the first, to our knowledge, to carry out a possible selves intervention in older, retired adults, in contrast to previous inquiries conducted in younger populations. Our research also proved innovative through its focus on physical activity identity in addition to physical activity, which has typically been the outcome of interest of physical activity possible selves interventions. Conducting the intervention entirely online was also novel as this, to the extent of our knowledge, was only the second time a possible selves intervention was delivered online [24]. Finally, in terms of design and analytic strengths, we assessed and controlled for personal characteristics (i.e., imaging ability, consideration for future consequences) and data were analyzed through mixed-effects modeling, which is increasingly advocated over traditional ANOVA-based techniques (e.g., [80]).

The strengths, however, must be considered in light of limitations. The demographics of participants (female, Caucasian, and well-educated) limit the generalizability of our findings. Further, our eligibility criteria pertaining to physical activity and identity allowed already fairly active retirees who somewhat identify as physically active to participate in the study, making it difficult for us to find effects. Another notable limitation concerns the use of a self-report physical activity measure which was predicated on practical considerations. Objective measurement was prohibitive given the online nature of the study. Further, we opted for the GLTEQ since it was previously validated against objective measures of physical activity and proven reliable in test-retest methods (for a review of these studies, please see [81]). Finally, the GLTEQ has been used with older adult/retired samples [20;21;36;57]. While our choice to use a validated measure of physical activity addresses some of the measurement limitations noted by Barnett et al. [12], our physical activity measure is limited in that it relies on self-report [82].

## Conclusion

This study was the first, to our knowledge, to test a possible selves intervention targeting physical activity identity and physical activity in a sample of recent retirees. While we did not find the expected differences between groups, marginal improvements in physical activity identity and behavior were observed over time (regardless of group assignment). We opine that interventions based on self and self-perceptions - like possible selves - may be relevant for new retirees (i.e., exploring/revisiting different roles), and as such, warrant further investigation. We hope our intervention can serve as a launch pad for future intervention efforts.

## Abbreviations

ANOVA: Analysis of variance
BMI: Body mass index
CFC: Consideration of future consequences
GLTEQ: Godin leisure time exercise questionnaire
IAQ: Imaging ability questionnaire
MCAR: Missing completely at random
URL: Uniform resource locator
